# TLR2 is non-redundant in the population and subpopulation responses to *Mycobacterium tuberculosis* in macrophages and *in vivo*

**DOI:** 10.1128/msystems.00052-23

**Published:** 2023-07-13

**Authors:** Charul Jani, Sydney L. Solomon, Joshua M. Peters, Stephanie C. Pringle, Amelia E. Hinman, Julie Boucau, Bryan D. Bryson, Amy K. Barczak

**Affiliations:** 1 The Ragon Institute of MGH, MIT, and Harvard, Cambridge, Massachusetts, USA; 2 Department of Biological Engineering, Massachusetts Institute of Technology, Cambridge, Massachusetts, USA; 3 The Division of Infectious Diseases, Massachusetts General Hospital, Boston, Massachusetts, USA; 4 Department of Medicine, Harvard Medical School, Boston, Massachusetts, USA; University of California Davis, Sacramento, California, USA

**Keywords:** tuberculosis, mycobacteria, macrophage, single-cell RNAseq, TLR2, innate immunity, *Mycobacterium tuberculosis*

## Abstract

Tuberculosis (TB), caused by the pathogenic bacterium *Mycobacterium tuberculosis* (Mtb), is a global health threat. Targeting host pathways that modulate protective or harmful components of inflammation has been proposed as a therapeutic strategy that could aid sterilization or mitigate TB-associated permanent tissue damage. In purified form, many Mtb components can activate innate immune pathways. However, knowledge of the pathways that contribute most to the observed response to live Mtb is incomplete, limiting the possibility of precise intervention. We took a systematic, unbiased approach to define the pathways that drive the earliest immune response to Mtb. Using a macrophage model of infection, we compared the bulk transcriptional response to infection with the response to a panel of Mtb-derived putative innate immune ligands. We identified two axes of response: an NF-kB-dependent response similarly elicited by all Mtb pathogen-associated molecular patterns (PAMPs) and a type I interferon axis unique to cells infected with live Mtb. Consistent with growing literature data pointing to TLR2 as a dominant Mtb-associated PAMP, the TLR2 ligand PIM6 most closely approximated the NF-kB-dependent response to the intact bacterium. Quantitatively, the macrophage response to Mtb was slower and weaker than the response to purified PIM6. On a subpopulation level, the TLR2-dependent response was heterogeneously induced, with only a subset of infected cells expressing key inflammatory genes known to contribute to the control of infection. Despite potential redundancies in Mtb ligand/innate immune receptor interactions during *in vivo* infection, loss of the TLR2/PIM6 interaction impacted the cellular composition of both the innate and adaptive compartments.

**IMPORTANCE:**

Tuberculosis (TB) is a leading cause of death globally. Drug resistance is outpacing new antibiotic discovery, and even after successful treatment, individuals are often left with permanent lung damage from the negative consequences of inflammation. Targeting host inflammatory pathways has been proposed as an approach that could either improve sterilization or improve post-treatment lung health. However, our understanding of the inflammatory pathways triggered by *Mycobacterium tuberculosis* (Mtb) in infected cells and lungs is incomplete, in part because of the complex array of potential molecular interactions between bacterium and host. Here, we take an unbiased approach to identify the pathways most central to the host response to Mtb. We examine how individual pathways are triggered differently by purified Mtb products or infection with the live bacterium and consider how these pathways inform the emergence of subpopulation responses in cell culture and in infected mice. Understanding how individual interactions and immune pathways contribute to inflammation in TB opens the door to the possibility of developing precise therapeutic interventions.

## INTRODUCTION

Tuberculosis (TB) caused an estimated 1.5 million deaths in 2021. New drugs that can tune the immune response to either better sterilize infection or reduce tissue pathology are needed to help end the ongoing global pandemic. Inflammation and individual inflammatory mediators can contribute to sterilization, pathology, or both, depending on the timing and magnitude of induction. A detailed understanding of the individual interactions central to the induced inflammatory response is a necessary step toward developing strategies to tune that response to achieve desired clinical outcomes.

Macrophages are the first cells infected and remain an important niche for the causative bacterium, *Mycobacterium tuberculosis* (Mtb), throughout the course of disease. Macrophage responses to bacteria are largely driven through interactions of host pattern recognition receptors (PRRs), which recognize pathogen-associated molecular patterns (PAMPs) or host cell damage associated with microbial or inflammatory responses. Mtb lacks a single-dominant PAMP, but multiple Mtb products have been described to function as PAMPs, inducing robust macrophage responses in their purified forms (1 - 8). Unbiased approaches to identify the most inflammatory components of the mycobacterial lipid repertoire have pointed to phosphatidylinositol dimannosides (PIMs) (9) and trehalose dimycolates (TDMs) (10, 11). However, it remains largely unknown which of these potential interactions are most critical for the composite response to infection with live Mtb, and whether, given redundancies in possible PAMP/PRR interactions driving inflammation, individual interactions make unique contributions to macrophage responses *ex vivo* and the cellular composition of responses *in vivo*. A strategy commonly employed by pathogenic bacteria is active interference with innate immune signaling pathway components to limit inflammation that may contribute to bacterial clearance. Whether the response induced by infection with live Mtb qualitatively and quantitatively approximates the response induced by individual purified Mtb products is also largely unknown.

Here, we sought to identify the molecular interactions that drive the earliest responses to Mtb on population and single-cell levels. Taking an unbiased approach, we found two dominant components of the macrophage response to infection with Mtb and identified the Mtb PAMP that induced the response most similar to infection with live Mtb. We then investigated the contribution of this dominant Mtb PAMP and its cognate PRR to the response to infection on population and subpopulation levels *ex vivo* and *in vivo*.

## RESULTS

### A panel of Mtb PAMPs induces qualitatively similar macrophage transcriptional responses

To identify the dominant molecular drivers of the macrophage response to Mtb, we first sought to define the transcriptional responses induced by purified Mtb products. In purified form, multiple Mtb products have been described to induce innate immune responses by activating a range of PRRs (1 - 8). We initially hypothesized that individual Mtb PAMP/PRR interactions would drive qualitatively unique transcriptional signatures that could then be distinguished as making unique contributions to the aggregate macrophage response to infection.

**Fig 1:**
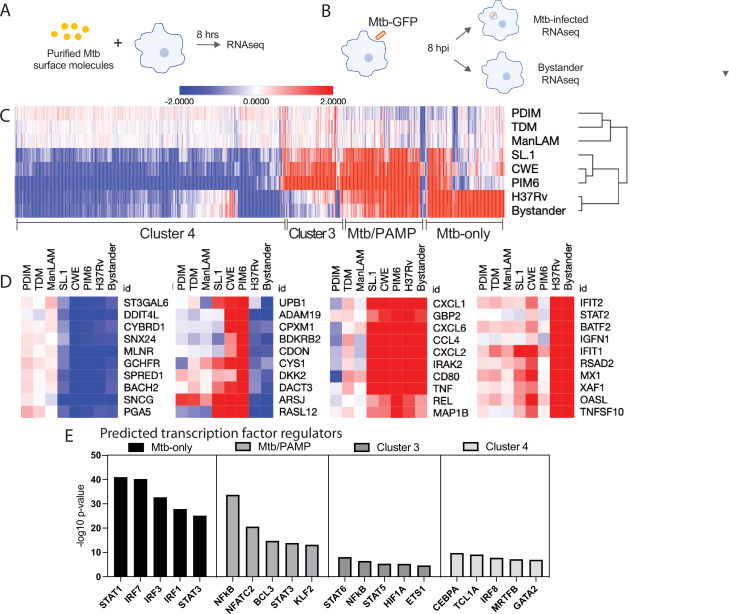
The macrophage response to Mtb infection is comprised of two dominant components. (**A and B**) Schematic overview of the experimental setup. PMA-differentiated THP-1 macrophages were treated with purified Mtb PAMPs for 8 h. For live Mtb infection, 4 h was permitted for phagocytosis. Eight hours post-phagocytosis, cells were sorted into GFP-positive Mtb-infected and GFP-negative bystander populations. RNA was extracted from each population and processed for RNAseq. (**C**) Heatmap showing hierarchical clustering of genes significantly altered (fold change >2, *q*-value < 0.05) by each stimulus compared to the untreated samples. (**D**) Representative genes from each cluster. (**E**) IPA was used to predict transcription factor regulators for each cluster.

To define the spectrum of responses to Mtb products, we profiled the transcriptional response of PMA-differentiated THP-1 cells to purified Mtb molecules proposed to function as PAMPs. Tested molecules included all putative Mtb PAMPs available through the NIAID-supported platform BEI Resources at the time of the initial experiment and included surface-exposed lipids and glycolipids [pthiocerol dimycocerosate (PDIM), TDM, and sulfolipid-1 (SL-1)], membrane-anchored mannan-based lipids [mannose-capped lipoarabinomannan (ManLAM) and phosphatidylinositol mannoside 6 (PIM6)] as well as total cell wall extract of Mtb strain H37Rv (TCWE). To optimize PAMP concentration and time post-exposure, we profiled the expression of Tumor necrosis factor (*TNF*), which is commonly used as a marker of PRR activation and has been used as an indicator of macrophage response to Mtb PAMPs (Fig. S1A and B). Based on these experiments and our previous work profiling macrophage transcriptional responses to Mtb infection (12), we selected standard treatment conditions for profiling the THP-1 cell response to each PAMP. We then treated PMA-differentiated THP-1 cells with each PAMP, harvested RNA, and performed comprehensive transcriptional profiling (Fig. 1A; Table S1).

Focusing on genes differentially expressed between conditions, we identified 902 genes changed at least twofold (log2 fold change ≥ 1, *q*-value ≤ 0.05) up or down in response to one or more PAMPs relative to expression in unstimulated THP-1 cells. Unsupervised hierarchical clustering of PAMPs based on gene expression patterns demonstrated that PAMPs fell into two groups (Fig. S1C). PIM6, SL-1, and TCWE induced the most significant transcriptional changes in terms of both number of genes changed and the magnitude of change. In contrast, TDM, PDIM, and ManLAM treatment elicited more modest transcriptional changes. However, the sets of genes induced or repressed were similar across PAMPs, suggesting that our hypothesis of qualitatively unique transcriptional signatures attributable to each PAMP/PRR pairing was incorrect. Instead, quantitative differences in response rather than distinct sets of regulated genes distinguished the macrophage response to different Mtb PAMPs.

### The macrophage response to Mtb is comprised of two dominant components

To determine how these qualitatively similar responses to individual Mtb PAMPs contribute to the aggregate macrophage response to Mtb, we next compared the PAMP-specific responses with the response elicited by infection with live Mtb. THP-1 cells were infected with Mtb expressing GFP (Mtb-GFP) at MOI 10:1. To permit separate transcriptional profiling of infected cells and bystanders (exposed, non-infected cells), cells were sorted into two populations: GFP-positive (Mtb-infected) and GFP-negative (bystander) (Fig. S1D); cells were profiled at 8-h post-infection to capture early transcriptional responses while minimizing cell death (Fig. 1B). Integration of Mtb-infected and bystander cells with PAMP-treated cells in expression analysis identified an additional 212 genes differentially regulated in the setting of Mtb infection. Hierarchical clustering analysis resulted in four gene clusters, each regulated by distinct sets of stimuli (Fig. 1C **;** Table S1). Genes upregulated by purified Mtb products separated into two clusters distinguished by their relative induction by Mtb. Genes in cluster 2 (“PAMP/Mtb”) were upregulated by Mtb PAMPs and by live Mtb (Fig. 1D) and included the cytokine *TNF*, which has been shown both experimentally and clinically to be critical to TB control (13, 14). *Il-1a* and *Il-1b*, which have been shown in mouse models to be critical for TB control (15), also fell into this cluster, as did the chemokines *CXCL1*, *CXCL2, CXCL6*, and *CCL4* (Fig. 1D). In contrast, expression of genes in cluster 3 was induced by Mtb PAMPs but either was not altered or was suppressed in the Mtb-infected and bystander populations (Fig. 1C and D). Cluster 1 (“Mtb-only”) was comprised of genes differentially regulated only in Mtb-infected and bystander cells (Fig. 1C and D). Cluster 4 was comprised of genes negatively regulated by live Mtb and/or one or more PAMPs (Fig. 1C and D). Visual inspection of genes in the “Mtb-only” cluster revealed multiple genes regulated by the type I interferon (IFN) response, including *IFIT1*, *IFIT2*, *RSAD2*, and *MX1* (Fig. 1D). Ingenuity pathway analysis (16) predicted type I IFN-associated regulators as upstream transcriptional factors (Fig. 1E). Visual inspection of “Mtb/PAMP” cluster genes revealed multiple genes regulated by NF-kB including *TNF*, *REL*, and *IRAK2* (Fig. 1D); IPA of this cluster predicted NF-kB as the highest-confidence upstream transcription factor regulator (Fig. 1E). Comparison with a recently published dataset demonstrated that murine homologs of a subset of genes in cluster 4 were downregulated in macrophages in a STING- and IFNAR-dependent fashion following infection with live Mtb (17).

### The type I IFN response comprises one dominant component of the macrophage response to Mtb and reflects microbial viability

**Fig 2:**
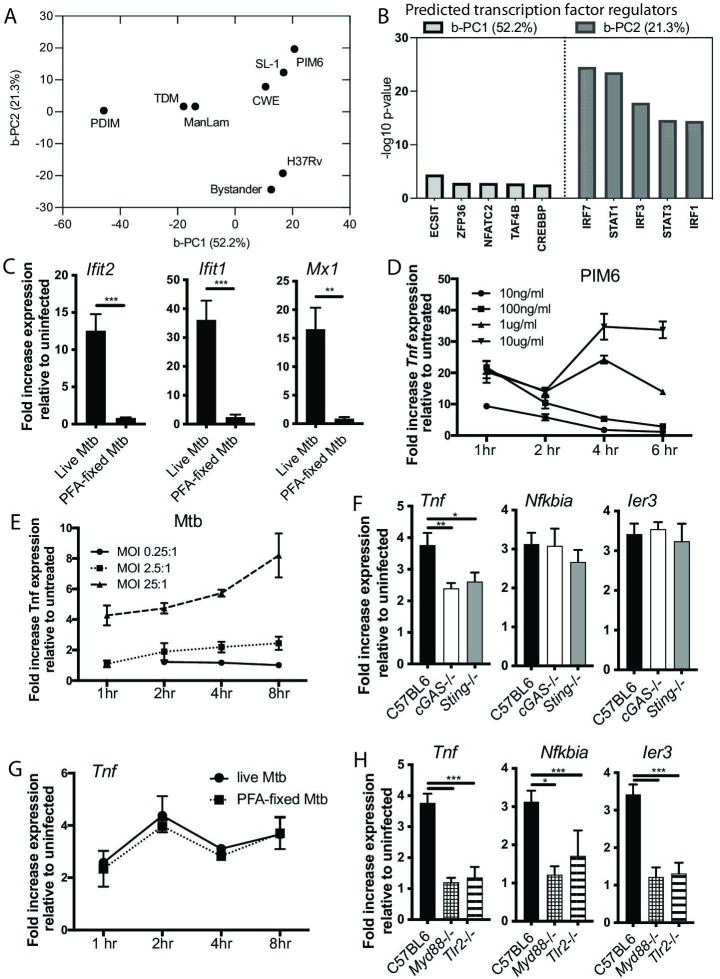
PIM6 and Mtb elicit qualitatively similar but quantitatively distinct macrophage responses. (**A**) Principal component analysis of eight conditions. b-PC1 (52.2% variance explained) was responsible for the majority of the differences between PAMPs. b-PC2 (21.3% variance explained) separated live *Mtb-*infected macrophages from all other conditions. (**B**) IPA was used to predict transcription factor regulators for b-PC1 and b-PC2. (**C and G**) C57BL6 bone marrow-derived macrophages (BMDMs) were infected with live-Mtb or paraformaldehyde (PFA)-inactivated H37Rv at an MOI of 5:1. RNA was harvested at 24 h (**C**) or at the indicated times post infection (**G**). (**D and E**) C57BL6 BMDMs were treated with PIM6 at the indicated concentrations (**D**) or infected with H37Rv at the indicated MOI (**E**), and RNA was harvested at the indicated timepoints. (**F and H**) The indicated BMDMs were infected with H37Rv at an MOI of 5:1. RNA was harvested 8 h post infection. Expression of the indicated genes was quantified by qPCR relative to GAPDH control. Mean ± SD; *P-value < 0.01, ** *P*-value < 0.001, ***P-value < 0.0001, unpaired two-tailed Student’s *t*-test.

To identify the dominant gene expression programs contributing to the observed differences across all conditions, we next turned to principal component analysis (PCA). PCA identified two principal components (PCs) that explained over 70% of the variance in the RNA sequencing data (Fig. 2A; Table S1). The first component, b-PC1, explained 52.2% of the variance (Fig. S2A); all conditions, including Mtb-infected, bystander, and all PAMPs treatments, were distributed along this axis (Fig. 2A). Comparison of the eigenvector values with expression values revealed that b-PC1 was composed predominantly of genes from the “PAMP/Mtb” cluster (Fig. S2B). The second principal component (b-PC2; variance explained, 21.3%) distinguished Mtb-infected and bystander cells from all PAMP treatment conditions (Fig. 2A). For b-PC2, comparison of eigenvector values with the expression values revealed that this component was predominantly comprised of “Mtb-only” cluster genes (Fig. S2B). Our results, thus, pointed to two dominant components of the macrophage response to infection with Mtb—a component induced by both infection with Mtb and treatment with Mtb PAMPs and a component that uniquely reflects infection with the live bacterium.

We next sought to identify regulators of the two dominant components of the macrophage response to Mtb. We first considered the Mtb-specific response component. As this component was comprised predominantly of genes in the “Mtb-only” cluster, we anticipated that similar regulators would be identified. Pathway analysis did, in fact, identify the type I IFN-associated factors STAT1, STAT3, IRF1, IRF3, and IRF7 as upstream regulators of b-PC2 (Fig. 2B). Consistent with the established model for induction of type I IFNs in Mtb-infected macrophages (18 - 20), induction of b-PC2 genes was dependent upon the PRR cGAS and upon STING (Fig. S2C). A subset of innate immune responses to bacterial infection are increasingly appreciated to reflect cellular detection of microbial viability (21, 22); in the context of macrophage infection with Mtb, the type I IFN response strictly depends on bacterial virulence functions, including ESX-1-mediated secretion (23). In our dataset, induction of type I IFNs uniquely distinguished infection with Mtb from all PAMP treatment conditions, consistent with a role for type I IFNs as reflecting microbial viability. Pre-treatment of Mtb with PFA, which inactivates the bacteria but leaves them structurally intact, led to loss of expression of “Mtb-only” cluster genes (Fig. 2C). These results, thus, suggested that the type I IFN response to Mtb comprises one dominant component of the macrophage response to Mtb and requires microbial viability.

### The NF-kB response to Mtb is a second dominant response component and is elicited in a qualitatively similar way by Mtb PAMPs

We next turned to the response component that was common to treatment with Mtb-purified PAMPs and infection with live Mtb. As this component was comprised largely of genes in the “Mtb/PAMP” cluster (Fig. S2B), we anticipated that predictions for upstream regulators would be similar. Consistent with this expectation, NF-kB pathway regulators ECSIT and TAF4B were predicted as upstream regulators of b-PC1 (Fig. 2B). Correlation analysis to identify the PAMPs eliciting the response most similar to Mtb infection demonstrated that Mtb-infected and bystander conditions were most strongly correlated with PIM6, TCWE, and SL-1 stimulation (Fig. S1E). PDIM, TDM, and ManLAM were grouped together and had a weaker correlation with the Mtb-infected and bystander conditions. Most of the tested PAMPs were positively correlated, with induced changes in the same direction. Interestingly, PAMP chemical structure or localization within the mycobacterial cell wall did not appear to dictate macrophage response, as PAMPs with structural similarities (such as Man-LAM and PIM6) or localization within a particular part of the bacterial cell wall (such as SL-1 and PDIM) did not necessarily cluster together. Our results indicated that the NF-kB response is a second dominant component of the response to Mtb infection that is qualitatively similar in the response to Mtb and a range of purified Mtb products.

### Within the shared component, infection with live Mtb elicits a slower, weaker macrophage response than exposure to purified Mtb products

We next asked whether the shared response component is induced in quantitatively similar or distinct ways following exposure to Mtb vs treatment with Mtb PAMPs. Although purified bacterial products can elicit highly inflammatory macrophage responses, the concentration, context of presentation, and context of recognition are substantially different in an intact microbe. Further, pathway interactions or active bacterial interference can limit the activation of inflammatory pathways. The TLR2 ligand PIM6 was one of the PAMPs most highly correlated with Mtb infection (Fig. S1E). Recent studies, including our own work exploring the interaction between Mtb virulence factors and effective inflammation, have pointed to TLR2 as central to the antibacterial response to Mtb (12, 24). To dissect the contribution of individual PAMP/PRR pairs to the macrophage response to Mtb, we, thus, selected PIM6/TLR2 for an in-depth study. *TNF,* which fell into the “PAMP/Mtb” cluster, was among the genes most heavily weighted in b-PC1 (Fig. 1D; Table S1); further, TNF was predicted as an upstream cytokine regulator of the “PAMP/Mtb” cluster, suggesting a role as a feed-forward positive regulator. Given the well-established practice of using *TNF* expression to quantify responses in PAMP/PRR studies and the importance of Tnf in clinical TB (13, 14)*,* we selected *TNF* expression as an output metric for our targeted testing.

To compare the kinetics and magnitude of responses to PIM6 and live Mtb, we profiled *Tnf* expression over a range of concentrations over time. Upon PIM6 stimulation, *Tnf* expression was rapidly and robustly induced (Fig. 2D). Consistent with the threshold effect previously described for macrophage responses to lipopolysaccharide (LPS) and our previous work with PAM3CSK4 (25, 26), *Tnf* expression was sustained in response to higher concentrations of PIM6 but waned over time in response to lower concentrations (Fig. 2D). In contrast, induction of *Tnf* expression in response to infection with Mtb was markedly slower and more modest (Fig. 2E). Even at high MOI, expression of *Tnf* was only eightfold induced after 8 hours, suggesting increased absolute concentration alone cannot overcome the quantitative differences between the response to live bacteria and purified PIM6.

We next considered factors that might contribute to quantitative differences in the response to live Mtb exposure vs PAMP treatment. Pathogens can interfere with the effective activation of innate immune signaling pathways through a variety of effector mechanisms (27, 28). In addition, pathway interference has been shown to restrict the full activation of the NF-kB-dependent response in other models of infection (29). We hypothesized that the relatively weaker, slower response to live Mtb might reflect (1) pathway interference between type I IFNs induced only by live bacteria and the NF-kB response (2), active bacterial subversion of pathway activation, or (3) the fundamental dynamics of PAMP recognition within the context of the mycobacterial surface. We reasoned that if type I IFN activation interferes with the induction of Mtb/PAMP genes, we would see more robust induction in cells unable to generate a type I IFN response. In fact, we found that expression of Mtb/PAMP cluster genes was similar between wild-type (WT) BMDMs and *cGAS* ^–/–^ or *Sting* ^–/–^ BMDM (Fig. 2F), excluding interference with the other dominant response component as a contributor to the weaker response to live Mtb. We then turned to the alternate hypothesis that active bacterial processes block the induction of NF-kB-dependent genes. We treated Mtb with PFA, exposed macrophages to equal ratios of live Mtb or PFA-treated Mtb, and then followed the transcriptional response over time. The response to PFA-inactivated Mtb, in fact, mirrored the response to live bacteria (Fig. 2G), excluding the possibility that active bacterial processes such as protein secretion interfere with a robust NF-kB response. We, thus, concluded that the slower, weaker response to Mtb likely reflected fundamental constraints on the recognition of PAMPs imposed by the context and concentration of PAMPs presented within the complex Mtb cell surface.

If PAMP context influences the kinetics and magnitude of PRR responses, we hypothesized that differences in kinetics and magnitude would be less pronounced for the comparison of abundant, highly inflammatory surface-localized PAMPs and the bacteria presenting them. We tested this hypothesis using the highly inflammatory PAMP LPS and the Gram-negative pathogen *Salmonella Typhimurium* (ST), which expresses LPS on its surface. We treated cells with increasing concentrations of LPS or infected with increasing MOI of ST and profiled *Tnf* expression over time. LPS robustly induced *Tnf* expression at all concentrations, with the timing of peak induction dependent upon concentration (Fig. S2E). The kinetics and magnitude of the response to live ST were highly similar to the response to LPS (Fig. S2F), suggesting that the nature of the PAMP influences the relative importance of PAMP context to the host response.

### The contribution of PIM6/TLR2 is non-redundant in the bulk macrophage response to Mtb

In contrast to our initial hypothesis that each PAMP would make qualitatively unique contributions to the macrophage response to Mtb, all Mtb PAMPs elicited qualitatively similar macrophage transcriptional responses. This observation led us to ask whether the PIM6/TLR2 interaction is, in fact, redundant with other PAMP/PRR pairs in inducing the aggregate macrophage response to Mtb. To study the unique contribution of PIM6/TLR2, we sought a genetic approach that would allow us to selectively remove the contribution of that interaction (schematic of known pathways tested, Fig. S3A). Deletion of Mtb genes in the PIM6 biosynthetic pathway could theoretically serve that purpose; however, PIM6 is an essential cell wall component, and mutants lacking PIM6 biosynthetic genes cannot be generated (30). We, thus, turned to host-side genetics, specifically a well-characterized *Tlr2*-knockout mouse (31). Induction of *Tnf* and other b-PC1 genes by PIM6 peaked 1–2 hours post-treatment in wild-type BMDM (Fig. S3B) and was entirely lost in *Tlr2* ^−/−^ BMDM (Fig. S3C), confirming that PIM6 is uniquely recognized by TLR2. Profiling expression of b-PC1 genes following Mtb infection in *Tlr2^−/^* ^−^ BMDM, we found that expression was reduced by approximately 50% (Fig. 2H). These results suggested that despite the potential redundancy of individual Mtb PAMPs for inducing the shared response component, individual PAMP/PRR interactions contribute quantitatively to the response to live Mtb in macrophages.

### Distinct subpopulations of macrophages drive the two dominant components of the response to live Mtb

**Fig 3:**
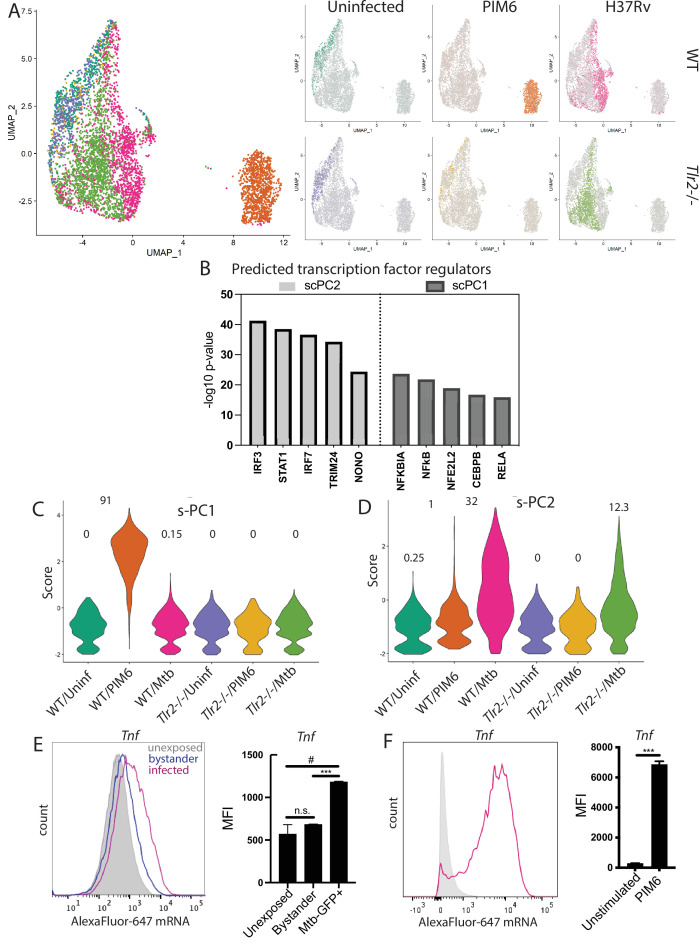
The two dominant components of the macrophage response to live Mtb exhibit different subpopulation dynamics. WT and *Tlr2^−^* ^/−^ C57BL6 BMDMs were treated with media, 1 μg/mL PIM6, or infected with H37Rv at an MOI of 2.5:1, and scRNA-seq was performed 8 h post infection. (**A**) Cells were projected onto a UMAP for visualization. (**B**) IPA was used to predict transcription factor regulators of single-cell principal component (s-PC)1 and s-PC2. (**C and D**) Cells were scored based on loadings of the top 50 positively contributing genes to (**C**) s-PC1 and (**D**) s-PC2. Numbers above each condition indicate the percent of cells in that condition scoring above 1 for each respective s-PC. (**E and F**) C57BL6 BMDMs were infected with H37Rv-GFP at an MOI of 2.5:1. Cells were fixed and processed according to the manufacturer’s protocol. Flow cytometry was then performed to assess fluorescently labeled transcript in GFP-positive (infected) and GFP-negative (bystander) cells relative to cells unexposed to Mtb. Histogram of flow plot for one representative replicates for each condition and mean fluorescent intensity (MFI) for unexposed, bystander, and infected cells for two biological replicates. Mean ± SD. ^#^*P*-value < 0.05, ***P-value < 0.0001, two-tailed unpaired *t*-test.

While innate immune responses have classically been studied in bulk populations, investigation at the level of individual cells has revealed that observed bulk responses are, in fact, comprised of heterogeneous subpopulation responses. Single-cell transcriptional profiling of ST-infected BMDMs has begun to elucidate the heterogeneity of macrophage responses to intact pathogens (32 - 34). Previous scRNAseq profiling suggested that multiple distinct responses can be observed in response to Mtb infection in human macrophages (35). We hypothesized that distinct subpopulation dynamics might contribute to the quantitative differences observed between macrophage responses to purified Mtb products and live Mtb. As a first approach to interrogating cell-to-cell variability and subpopulation dynamics, we employed scRNAseq; scRNAseq offers an entirely unbiased approach to defining cellular subpopulations based on dominant transcriptional programs but does not capture bacterial RNA and, thus, cannot distinguish Mtb-infected from uninfected cells in a mixed population. We utilized the 10× Genomics platform with sample barcoding to profile the transcriptional response to Mtb and PIM6 in WT or *Tlr2* ^−/−^ BMDM. After sample processing, sequencing, and demultiplexing, gene expression signatures were projected into a two-dimensional visualization using Uniform Manifold Approximation and Projection (UMAP) (36). Visualizing these samples via UMAP revealed that WT unstimulated, *Tlr2* ^−/−^ unstimulated, and *Tlr2* ^−/−^ PIM6-treated cells did not separate, suggesting that WT and *Tlr2* ^−/−^ cells were in transcriptionally similar states at baseline and affirming our bulk finding that the transcriptional response to PIM6 is uniquely dependent upon TLR2 (Fig. 3A). PIM6-stimulated WT BMDM largely separated from all other samples. Mtb-exposed WT and Mtb-exposed *Tlr2* ^−/−^ cells separated from unexposed cells and partially separated from one another, suggesting some cells in transcriptionally similar states and other cells in transcriptionally distinct states. A small subset of Mtb-exposed WT BMDM was directly overlaid on the PIM6-stimulated condition, indicating that only a few Mtb-exposed WT BMDM were in a transcriptional state similar to that induced by PIM6. Cells in the Mtb-exposed *Tlr2^−^* ^/−^ BMDM condition fully separated from PIM6-treated WT BMDM cells, suggesting that in the absence of TLR2, no Mtb-exposed cells are in a transcriptional state similar to that induced by PIM6 stimulation. In aggregate, these results suggested that differences in elicited subpopulations contribute to observed differences between PAMP-treated and Mtb-exposed cells.

To define the transcriptional programs governing single-cell responses, we next performed PCA of the scRNAseq data. PCA (s-PCA) revealed two dominant response components, with many additional components making small contributions (Fig. S4A and B) as is common for single-cell datasets. Analysis of the top 50 genes contributing to the dominant s-PCs demonstrated no overlap between genes contributing to s-PC1 and s-PC2 and very little overlap between s-PC1 or s-PC2 and the other s-PCs through s-PC5 (Fig. S4C). Predicted regulators of s-PC1 included multiple NF-kB-related transcription factors (Fig. 3B), consistent with predictions for our bulk data. Predicted regulators of s-PC2 included IFN-dependent transcription factors (Fig. 3B), also consistent with bulk data predictions. These results indicated that similar to our bulk analysis, NF-kB-dependent gene expression and Type I IFN-dependent gene expression are the two dominant axes of macrophage response to Mtb exposure on a single-cell level.

We next considered how homogeneously each response component was induced in each condition. Scoring each condition based on the expression of s-PC1 genes, we found that expression was induced in almost all PIM6-stimulated wild-type BMDM (Fig. 3C; Fig. S4D). In contrast, significant expression of s-PC1 genes was induced in only a small subset of Mtb-exposed WT BMDM. Distinct from WT BMDM, s-PC1 genes were not induced in Mtb-exposed *Tlr2* ^−/−^ BMDM, suggesting that individual PAMP/PRR interactions are non-redundant on a single cell level but instead may drive the emergence of defined subpopulations of responding cells. As expected, there was no induction of s-PC1 genes in unstimulated WT or *Tlr2^−^* ^/−^ BMDM or in PIM6-treated *Tlr2* ^−/−^ BMDM. In contrast to s-PC1, s-PC2 genes were more homogeneously induced across Mtb-exposed cells (Fig. 3D; Fig. S4E). Expression was slightly less in Mtb-exposed *Tlr2^−^* ^/−^ BMDM than WT BMDM. Thus, while the type I IFN response, which has been associated with disease progression, was induced across a larger proportion of Mtb-exposed cells, the NF-kB component, which has been classically associated with disease control, was induced in only a small proportion of Mtb-exposed cells. Together, our data support a model in which distinct transcriptional programs are induced in subsets of Mtb-exposed cells, with only a small proportion of exposed cells mounting the type of robust NF-kB-dependent response that could be induced by exposure to purified Mtb ligand.

### The shared response component is incompletely induced in infected macrophages

Because PIM6 is a soluble factor, all PIM6-exposed cells are likely similarly exposed to PIM6, while only a fraction of Mtb-exposed cells will be actively infected. However, not all Mtb-exposed cells take up the bacterium; we reasoned that infected and bystander cells likely differ in their PAMP exposure. Because our scRNAseq analysis did not distinguish actively infected cells, we hypothesized that the infection status of cells contributed to the heterogeneity of responses observed. In addition, while scRNAseq is useful for identifying subpopulation responses, the depth to which individual cell transcript is captured is limited and may miss genes expressed or induced at relatively low levels. To distinguish transcriptional responses in individual infected and bystander cells and more completely capture selected transcripts in individual cells, we turned to the complementary single-cell approach of Flow Fluorescent In-Situ Hybridization (FlowFISH) (37). We modified the protocol to ensure that processing did not alter the GFP signal from Mtb (Fig. S5A). We then infected cells with GFP-expressing Mtb and profiled the expression of *Tnf*. Of the 35%–40% of cells infected with Mtb, 78% expressed *Tnf* with relatively low induction of transcript (an average 4.6-fold increase in MFI relative to cells unexposed to Mtb) (Fig. 3E; Fig. S5B and C). Thus, 22% of Mtb-infected cells had no appreciable induction of *Tnf* transcript. In bystander cells, only 47% expressed *Tnf* transcript, with an average 1.9-fold increase in MFI (Fig. 3E). In total, 53% of all cells exposed to Mtb expressed *Tnf* transcript, with higher expression induced in infected cells. In contrast, 84% of PIM6-exposed cells expressed *Tnf* transcript with a 13.6-fold increase in MFI (Fig. 3F). These results suggested that both the proportion of responding cells in a population and the relative response of each responding cell distinguish the response to PIM6 vs infection with live bacteria. These results support a model in which subpopulation dynamics distinguish host cell responses to PAMPs from responses to live bacteria, with distinct response phenotypes in infected and bystander cells within the exposed population contributing to subpopulation differences.

### The TLR2/PIM6 interaction is non-redundant *in vivo* and drives the development of a distinct infection microenvironment

**Fig 4:**
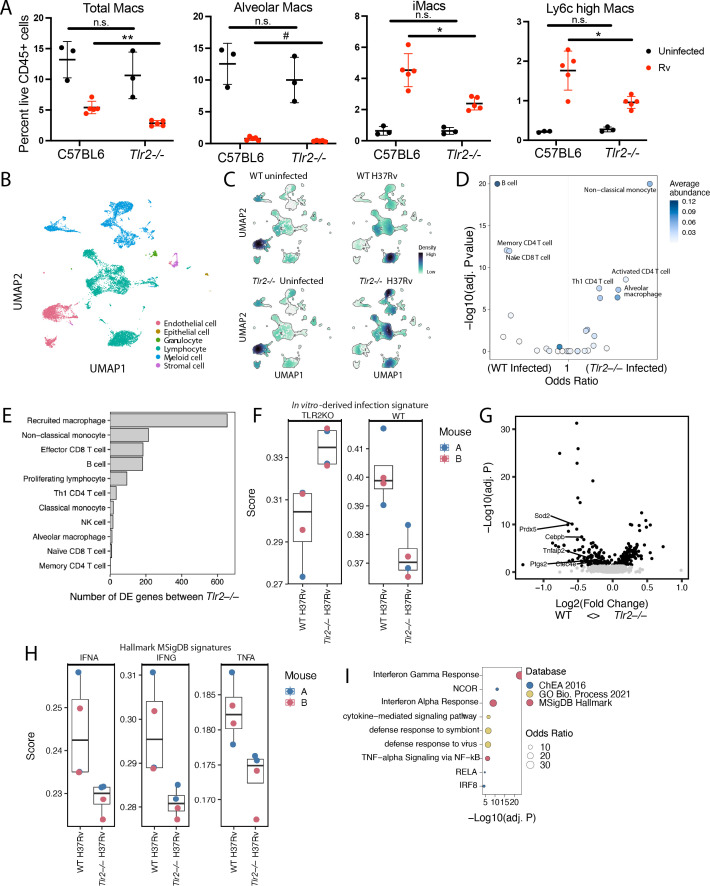
TLR2 shapes the cellular microenvironment *in vivo* in the Mtb-infected lung. C57BL6/6J or *Tlr2^−^* ^/−^ mice were infected with Mtb (~200 CFU). Six-week post infection, mice were euthanized, lungs were harvested, and single-cell suspensions were generated for flow cytometry analysis and scRNAseq. (**A**) Flow cytometry analysis of total macrophages and macrophage subpopulations as a percent of live CD45+ cells (3–5 biological replicates per group). (**B–H**) scRNAseq (two biological and two technical replicates). (**B**) UMAP projection of cell type lineages. (**C**) Cell density projection on UMAP projections for each condition (WT uninfected, WT infected, *Tlr2* ^−/−^ uninfected, *Tlr2* ^−/−^ infected). (**D**) Odds ratio and FDR-adjusted *P* value for differential abundance changes between infected WT and *Tlr2* ^−/−^ samples calculated using a generalized binomial linear model. (**E**) Number of differentially expressed genes (DE genes) within each cell type between infected WT and *Tlr2^−^* ^/−^ mice. (**F,** and **G**) Volcano plot describing fold change and FDR-adjusted *P* values for DE genes in recruited macrophages between infected WT and *Tlr2^−^* ^/−^ mice. Labeled genes are among the genes that contribute most significantly to s-PC1. Average infection signature scores between WT and *Tlr2^−^* ^/−^ mice within recruited macrophages. (**H**) Average Hallmark MSigDB signature scores between WT and *Tlr2^−^* ^/−^ mice within recruited macrophages. (**I**) Enrichment analysis of DE genes in WT recruited macrophages across three distinct databases.

In macrophage culture, we found that the PIM6/TLR2 interaction made a unique contribution to the response to Mtb on both bulk and single-cell levels. However, while early macrophage responses to infection in cell culture reflect cell autonomous activation of signaling pathways and paracrine effects from neighboring identical cells, the macrophage response *in vivo* arises within a substantially more complex microenvironment. Contributors to that complexity include a range of neighboring immune and non-immune cells, the immediate cytokine milieu, and interactions with matrix. In the homogeneous environment of BMDM in cell culture, only a subpopulation of infected cells activated a TLR2-dependent inflammatory program upon infection (Fig. 3A). We hypothesized that in the substantially more complex environment of the Mtb-infected lung, any unique contribution of TLR2 to the evolving host response might be entirely lost given potential redundancy in PAMP/PRR interactions. In this case, we would expect responses in wild-type and *Tlr2^−^* ^/−^ mice to be indistinguishable. Alternatively, we hypothesized that *Tlr2*-dependent signal in a subset of infected macrophages *in vivo* might drive evolution of a unique microenvironment, amplifying differences we had observed between WT and *Tlr2^−^* ^/−^ BMDM both within and beyond the myeloid compartment. To distinguish between these possibilities, we sought to define the cellular composition and transcriptional states of cells from the lungs of wild-type and *Tlr2* ^−/−^ mice. We infected WT and *Tlr2^−^* ^/−^ C57BL/6J mice (*n* = 2 per strain with two technical replicates per mouse) with aerosolized *Mtb* H37Rv (Fig. S6A). At six weeks post infection, we harvested lungs for scRNAseq and flow cytometry to quantify cellular subsets and identify subpopulations of cells based on transcriptional states. By flow cytometry, differences between WT and *Tlr2* ^−/−^ mice were most notable in macrophage subsets (Fig. 4A; Fig. S6B). These results suggested that the TLR2-dependent response is not subsumed *in vivo* by redundant PAMP/PRR interactions. Given that CFU were similar at week 6 post infection in wild-type and *Tlr2^−^* ^/−^ mice, observed differences in cellular influx did not simply represent responses to different bacterial burdens (Fig. S6A).

To take a more in-depth approach to defining the impact on individual PAMP/PRR interactions on the evolution of cellular subsets, we next performed scRNAseq. Profiling roughly 20,000 cells split across uninfected and infected WT and *Tlr2* ^−/−^ mice, we identified approximately 30 cell types across major immune and structural lineages (Fig. 4B; Fig. S7A through C). Populations of cells identified included memory CD4+ T cells, CCR7+ mature regulatory dendritic cells (mregDCs), and recruited macrophages (C1qb+). After cell annotation, we performed differential abundance analysis using a generalized binomial linear model, which suggested significant differences in the cellular compositions in the lungs of Mtb-infected *Tlr2* ^−/−^ and WT mice (Fig. 4C and D). Recent work using scRNAseq as a tool to look at the relationship between myeloid cell subpopulations and bacterial state had defined subsets of myeloid cells present in mouse lung 3 weeks post infection (38). Comparing the cellular subsets we identified with in that work, we found excellent overlap overall in cellular classifications (Fig. S7D). Three subtypes of interstitial macrophages from that work we classified as recruited macrophages, while a fourth subtype we classified as monocyte-derived dendritic cells (Fig. S7E). Two of their identified alveolar macrophage subsets we classified as alveolar macrophages, with a third classified as proliferating alveolar macrophages and a fourth as myeloid cells (Fig. S7E). Similar to that work, we found that infection drove strong immune cell infiltration into the lung. Examining how the presence of TLR2 impacted cell recruitment, we found that several immune populations were differentially abundant between infected *Tlr2* ^−/−^ and WT mice. Although expression of TLR2 is predominantly restricted to the myeloid population, the fractional abundance of other cell types, including distinct T cell subpopulations, differed between wild-type and *Tlr2* ^−/−^ mice (Fig. S7F and G). These results suggested that TLR2-dependent responses make a unique contribution *in vivo* and, in fact, that contribution is amplified beyond cells that themselves express TLR2.

Given that loss of TLR2 changed the relative abundance of cellular subsets in infected mice, we next sought to define transcriptional differences within cell populations of these mice. We performed differential gene expression analysis per cell type which revealed strong differences within the recruited macrophages and non-classical monocyte populations specifically, followed by various lymphocyte populations (Fig. 4E). We then focused on the recruited macrophage population to characterize these transcriptional differences. First, we asked if the differences seen *in vitro* between infected *Tlr2^−^* ^/−^ and WT BMDMs (as shown in Fig. 3) are manifested *in vivo*. We scored the *in vivo* recruited macrophages using the gene sets we had identified as upregulated in each *in vitro* population (Table S2). Genes upregulated in infected WT BMDM were more expressed in infected WT mice, and conversely genes upregulated in infected *Tlr2^−^* ^/−^ BMDM were more expressed in infected *Tlr2^−^* ^/−^ mice. These results suggested that differences observed in cultured macrophages continue to manifest to some extent *in vivo* as far as six weeks post infection (Fig. 4F and G). We additionally scored recruited macrophage populations using mouse orthologs of the MSigDB Hallmark gene lists for TNFα, IFNγ, and IFNα responses, revealing increased responses for all three gene sets in infected WT mice relative to infected *Tlr2^−^* ^/−^ mice (Fig. 4H). Lastly, we identified the top genes upregulated in WT-recruited macrophages and performed an enrichment analysis using the ChEA (2016), GO Biological Process (2021), and MSigDB databases in Enrichr (Fig. 4I). As expected, all three Hallmark gene sets were enriched in addition to RELA and IRF8. NCOR was also enriched, a transcriptional checkpoint for macrophage activation among other signaling programs. All together, these data suggest that the earliest TLR2-dependent transcriptional responses observable in macrophages infected *in vitro* also occur *in vivo* and impact the lung microenvironment up to at least 6 week post infection. More broadly, these data suggest that in spite of the number of Mtb PAMPs and consequent potential redundancy for inducing host responses, individual PAMP/PRR interactions contribute uniquely to the lung microenvironment, impacting both the cellular subsets that emerge post infection and the transcriptional states of those cells.

## DISCUSSION

“Tuning” the inflammatory response in TB infection has been proposed as a strategy for improving treatment outcomes; to date, approaches taken have largely focused on relatively non-specific, broadly active immune modulators (39). A detailed understanding of the contribution of individual pathways to host inflammation upon infection with Mtb would enable more precise interventions and tailored to clinical presentation or host phenotype. Distinct from Gram-negative pathogens, for which a dominant, highly inflammatory surface PAMP drives the inflammatory response, Mtb contains a spectrum of subdominant PAMPs (1 - 8), not all of which are readily surface-accessible. This complexity and potential redundancy make it challenging to identify the pathways most important during infection, thus offering the most promising targets. Here, we took an unbiased approach to identify the molecular interactions that contribute most to the inflammatory response to Mtb; the PIM6/TLR2 interaction emerged as driving a response most qualitatively similar to a predicted NF-kB-dependent component of the response to live Mtb.

Our findings of the centrality of TLR2 in the macrophage response to Mtb are consistent with recent work studying the interactions of live Mtb with host macrophages. In models using reporter cell lines or purified Mtb products as stimuli, many Mtb components have been shown to elicit inflammatory responses; distinguishing which of many potential interactions contribute to the aggregate response to the live bacterium and to what extent redundancy renders individual interactions moot is not possible using minimalist models. In recent work using an Mtb transposon mutant library to identify mycobacterial factors that interfere with NF-kB activation, Mtb mutants impaired in production of the glycolipid SL-1 induced more robust NF-kB signaling in a TLR2-dependent fashion (24). This work identified TLR2 as having the potential to recognize and respond to the intact bacterium but raised the possibility that TLR2 makes a limited contribution to the overall response to live Mtb because of lipid interference with effective pathway activation. In previous work, we identified a late, endosome-specific component of the TLR2 response as blunted by the phagosomal membrane damage carried out by key Mtb virulence factors (12). Similar to the findings in Blanc et al., our work pointed to TLR2 but raised the possibility that the contribution of TLR2 to the overall response is limited by multi-pronged mycobacterial interference. Our findings here suggest that, in spite of mycobacterial efforts to limit TLR2 activation, interactions between TLR2 and Mtb TLR2 ligands are a dominant contributor to NF-kB-dependent responses to Mtb. While Man-LAM was identified as the first Mtb-derived TLR2 ligand (40), consistent with other work (41), we find that PIM6 is a more robust TLR2 agonist. This work is additionally consistent with unbiased work identifying PIMs as the most inflammatory component in Mtb lipid microspheres (10, 11). It remains uncertain which TLR2 agonist or agonists are most dominant *in vivo*, and it is likely that multiple agonists contribute to the TLR2-dependent signal.

While we identify PIM6 as eliciting an inflammatory response qualitatively similar to the macrophage response to live Mtb, our results identify quantitative differences in the responses elicited by the two stimuli. We found that purified PIM6 could elicit as rapid and robust an inflammatory response as the highly inflammatory, dominant PAMP LPS. However, infection with live bacterium resulted in a slower, weaker response. The slower kinetics and lower magnitude could not be overcome by either increasing bacterial concentration or inactivation, excluding the possibilities that limited total PAMP concentration or active bacterial processes constrain the response. Together with our previous work suggesting a threshold for sustained TLR2 activation following stimulation with synthetic ligand (26), these results raise the possibility that the local concentrations and/or context of Mtb PAMPs within the live bacterium may limit overall activation of TLR2. Previous work explored in detail how context and presentation of one biologically important Mtb lipid, TDM, changes the recognition and inflammatory properties of the lipid (42 - 45). Our work is consistent with these findings and suggests that context and presentation likely influence recognition of and subsequent host response to a range of Mtb PAMPs. While our focus in this work was exclusively on the dominant innate immune signaling pathways activated by Mtb upon infection, the broader literature suggests that Mtb products may limit additional TLR2-dependent defenses, including activation of autophagy (46).

Our results additionally demonstrate significant heterogeneity in induction of the dominant inflammatory response in macrophages. On a single-cell level, we found that the dominant response components were incompletely induced in Mtb-exposed or Mtb-infected cells, with only a small population of cells expressing a robust NF-kB-dependent response similar to purified PAMP when profiled by scRNAseq and incomplete induction of *Tnf* in infected cells when studied using FlowFISH. Any exposure to Mtb resulted in inflammatory responses distinct from unexposed cells; the identity of cells as infected or bystanders was one main driver of differential response within exposed populations. The extent to which released cytokines vs cell-to-cell release of mycobacterial products (47) influence bystander phenotypes is unknown. Further, our studies, which require cell fixation or harvest, necessarily offer only a snapshot of the total response. TNF has been described to have dual roles in infection—some TNF is critically important for Mtb control in experimental models and in clinical studies (13, 14), but too much TNF drives macrophage necrosis and release of bacteria to infect new cells (48). Our findings of heterogeneous induction of *Tnf* and co-regulated genes raise the question of whether foci of progressive infection can ultimately be traced back to subsets of macrophages with a relatively anemic NF-kB-dependent response to infection. We anticipate that emerging technologies enabling tracking over time of individual infected host cells with defined phenotypes will ultimately allow questions of how subpopulations of cells differentially contribute to disease outcomes to be asked and answered.

Heterogeneity is, in fact, a hallmark of clinical TB (49 - 51). The capacity to phenotype individual cells within complex populations has opened the door to understanding how cellular phenotypes contribute to TB disease complexity within the host environment. Two recent efforts have used scRNAseq to profile cells within established granulomas. In a study using both zebrafish and macaque models of mycobacterial infection, Type 2 activation and Stat6 were found to drive formation of necrotic granulomas (52). In a parallel study in macaques comparing the cellular composition of granulomas that are PET-apparent by 4 week post infection (“early granulomas”) with those that are not apparent until 10 week post infection, early granulomas had a stronger Type 2 signature and higher bacterial burdens (53). These snapshots of granuloma composition suggest cellular correlates of bacterial control and failure to control after the disease is established. A complementary study using scRNAseq and cyTOF to define subpopulations of cells in the lung that uniquely distinguish latent and active TB in a macaque model suggested cellular subsets that may contribute to control and failure to control infection (54). Our results suggest that heterogeneity in the host response to TB infection is not only introduced at the level of established granuloma or whole-organism infection but also, in fact, encoded from the very first encounter between individual macrophages and infecting mycobacteria. The full sequence of events linking the subpopulation responses we observe in macrophages with the recruitment of distinct cellular populations and the formation of granulomas with differing capacities to control infection remains to be revealed. Our *in vivo* murine analyses highlight the importance of PRR engagement in orchestrating the complex multi-cellular response to infection. Previous studies in mice lacking TLR2 have sought to determine how loss of this PRR contributes to overall bacterial burden; however, these diverse studies (Mtb strain of infection, infection dose) arrive at distinct conclusions that make it difficult to illuminate common features of immunity in mice lacking TLR2 (55 - 58). Our studies reveal that loss of TLR2, which is largely restricted to myeloid cells, contributes to alterations in the abundance of non-classical monocytes, T cells, and B cells, suggesting that the overall quality of the adaptive immune response in these animals may be different. How these altered adaptive immune cell dynamics in the absence of TLR2 signaling contribute to immunopathology and the generation and maintenance of antigen-specific T cell responses are important areas for future inquiry. Together with recent work suggesting that the bacterium actively subverts TLR2 activation, our results suggest that modulating the TLR2 pathway, including strategies to collapse heterogeneity within populations of infected cells, may offer precision targets for future host-directed TB therapeutics.

## MATERIALS AND METHODS

### Isolation of bone marrow-derived macrophages

All animal use protocols were approved by the MGH IACUC and carried out in accordance with national guidelines for the ethical use of animals in research. C57BL/6J (Jackson Laboratories strain [Bar Harbor, ME, USA] Number 000664), *Tlr2* ^−/−^ (B6.129-*tlr2^tm1Kir^*/J, Jackson Laboratories strain number 004650), *Sting* ^−/−^ (C57BL/6J-*Sting^gt^*/J, Jackson Laboratories strain number 017537), and *cGAS* ^−/−^ (B6(C)-Cgas^tm1d(EUCOMM)Hmgu^/J, Jackson Laboratories strain number 026554) mice were ordered from Jackson Laboratories. Mice were euthanized by carbon-dioxide inhalation, and femurs and tibias were harvested for bone marrow isolation. Bone marrow cells were incubated at 37°C with 5% carbon dioxide in BMDM media (DMEM [Gibco, Billings, Montana, USA] with 20% fetal bovine serum [Hyclone, Logan, Utah, USA] and 25 ng/mL recombinant mouse M-CSF [R and D Systems, Minneapolis, MN, USA]) on petri dishes. After 6 days, adherent cells were washed and harvested for use as bone marrow-derived macrophages.

### Cell culture

The indicated *Mtb* strains (H37Rv and H37Rv-GFP) were grown in Middlebrook 7H9 broth (Difco) with Middlebrook OADC (BD), 0.2% glycerol, and 0.05% Tween-80. THP-1 monocytes were grown in R10 media (RPMI-1640 supplemented with 0.5 mM 2-mercaptoethanol and 10% FBS [Hyclone, Logan, Utah USA]). THP1 cells were differentiated in R10 media containing 25 ng/mL PMA for 24 h. Cells were then washed with PBS twice and incubated for 24 h in fresh R10 media for recovery prior to use in experiments. BMDMs were grown overnight in BMDM media prior to treatments or infections.

### PAMP treatment and Mtb infections

The purified Mtb surface molecules were obtained from BEI Resources (PDIM: NR-20328, PGL: NR-36510, TDM NR-14844, LAM: NR-14848, PIM2: NR-14846, PIM6: NR-14847, SL-1: NR-14845) and resuspended in DMSO at 1 mg/mL. DMSO carrier was used as the comparator control. Mtb infections were carried out as previously described (59, 60). Briefly, *Mtb* strain H37Rv was grown to mid-log phase, washed once in PBS, resuspended in PBS, and subjected to a low-speed spin to pellet clumps. Macrophages were infected at the indicated MOI, allowing 3–4 h for phagocytosis. Cells were then washed once with PBS, and media were added back to washed, infected cells. For paraformaldehyde fixation, Mtb was pelleted by centrifugation and then resuspended in 4% paraformaldehyde for 1 h at room temperature. Cells were then pelleted by centrifugation, washed twice in PBS, and resuspended in PBS.

### RNA extraction and qPCR

Infected or treated BMDM/THP-1 were lysed at designated time points with β-ME-supplemented Buffer RLT (Qiagen). RNA was isolated from lysate using an RNEasy kit (Qiagen) supplemented with RNase-free DNase I digest (Qiagen), both according to manufacturer’s protocol. cDNA was prepared using SuperScript III (Thermo Fisher Scientific, Waltham, MA, USA) according to manufacturer’s protocol. qPCR was performed using PowerUP SYBR Green (Thermo Fisher Scientific, Waltham, MA USA) and primers specific to investigated genes relative to *Gapdh* control. Primers sequences used for qPCR: mouse *Irak2*: F-GAAATCAGGTGTCCCATTCCAG and R-TGGGGAGGTCGCTTCTCAA; mouse *Traf1*: F-TCCTGTGGAAGATCACCAATGT and R-GCAGGCACAACTTGTAGCC; mouse *Nfkbia*: F-CTCCGAGACTTTCGAGGAAATAC and R-GCCATTGTAGTTGGTAGCCTTCA; mouse *Ifit1*: F-CTGAGATGTCACTTCACATGGAA and R-GTGCATCCCCAATGGGTTCT; mouse *Mx1*: F-GACCATAGGGGTCTTGACCAA and R-AGACTTGCTCTTTCTGAAAAGCC; mouse *Ifit2*: F-CGAGCAGACAGTTACACAGCAGTCA and R-CGTTGGCATTTTAGCTGTCGCAGAT; mouse *Gapdh*: F-CGACCCCAACACTGAGCATCTCC and R-CGTCCCTAGGCCCCTCCTGTTATTAT; mouse *Ier3*: F-CGACCAGCTACCAACCGAGGAA and R-TCGGAAAGAGGACCCTCTTGGCAA; mouse *Tnf*: F-CGAGCCTCTTCTCATTCCTGCTTGTG and R-CGTTCATCCCTTTGGGGACCGATC.

### Bulk RNA-Seq

Poly(A)-containing mRNA was isolated from 1 µg total RNA using NEBNext Poly(A) mRNA Magnetic Isolation Module (New England Biolabs, Ipswich, MA, USA). cDNA libraries were constructed using NEBNext Ultra II Directional RNA Library Prep Kit for Illumina and NEBNext Multiplex Oligos for Illumina, Index Primers Sets 3 and 4 (New England Biolabs, Ipswich, MA USA). Libraries were sequenced on an Illumina NextSeq500. Bioinformatic analysis was performed using the open source software GenePattern (55, 61). Raw reads were aligned to mouse genome using TopHat, Cufflinks was used to estimate the transcript abundance, and Cuffdiff was used to calculate fold difference in expressions and the log2 fold change values (with *P-*value ≤ 0.05 and *q*-values ≤ 0.05) were used to plot the heatmap. Correlation analysis, principal component analysis, cluster analysis, and visualization were performed in RStudio and Morpheus (https://software.broadinstitute.org/morpheus). Functional analysis was performed using IPA (Qiagen Inc., https://www.qiagenbio-informatics.com/products/ingenuity-pathway-analysis).

### FlowFISH/PrimeFlow assays

PrimeFlow RNA Assay Kit (Thermo Fisher; Catalog number: 88-18005) was used to stain for *Tnf*, (probe ID number VB1-10175-PF) and control *Rpl13a* (probe ID number VB6-15315-PF), according to the manufacturer’s instructions with several modifications. Specifically, the permeabilization of infected macrophages was performed in ice-cold methanol for at least 15 min instead of permeabilization buffer supplied. The permeabilized cells were treated with 2% PFA in PBS and washed twice with the wash buffer. Cells were then incubated with the hybridization probes as indicated, and rest of the staining was performed as per the manufacturer’s instructions.

### Mouse infections

All mouse experiments were carried out under protocols approved by the Massachusetts General Hospital Institutional Animal Care and Use Committee. Seven- to eight-week-old female C57BL/6J (Jackson Laboratories strain number 000664) or *Tlr2* ^−/−^ (Jackson Laboratories strain number 021302) mice were infected via low-dose aerosol exposure with an AeroMP (Biaera Technologies, Hagerstown, MD, USA). Three to five mice per condition were harvested at day 0 to quantify inoculum. Six weeks post infection, mice were euthanized in accordance with AALAC guidelines, and lungs were harvested for histopathology, CFU, and tissue dissociation for scRNA-seq and flow cytometry quantification of cell subsets.

### Murine lung cell flow cytometry

After harvest, murine lungs were dissociated using a GentleMACS Dissociator (Miltenyi Biotec, Bergisch Gladbach, Germany) in digestion buffer (RPMI with 10 mM HEPES, DNAse I 50 μg/mL, Liberase TM 100 μg/mL, and 2% FBS). After running the m_lung_01 program, samples were incubated at 37°C for 30 min before running the m_lung_02 program. Samples were filtered with a 70-μM filter, washed once, and then RBCs were lysed for 5 min using RBC Lysis Buffer (Sigma-Aldrich, Burlington, MA, USA). Samples were then quenched with FACS buffer (PBS with 2% FBS and 2 mM EDTA) and washed once. Cells were stained with fixable viability dye eFluor 455UV (Invitrogen, Carlsbad, CA, USA), incubated with Fc receptors block (TruStain FcX, clone 93, BioLegend, San Diego, CA, USA), and stained with a panel of immunophenotyping antibodies at room temperature for 30 min. The panel was made of the following antibodies (clone, dilution, manufacturer): CD45 BUV395 (30-F11, 1:400, BD Biosciences, San Jose, CA, USA), CD24 BV510 (M1/69, 1:500, BioLegend), I-A/I-E Pacific Blue (M5/114.15.2, 1:1200, BioLegend), CD64 Pe/Cyanine7 (X54-5/7.1, 1:50, BioLegend), CD11c PerCP (N418, 1:200, BioLegend), CD11b (M1/70, 1:1500, BioLegend), Ly-6G BV605 (1A8, 1:1500, BioLegend), Ly-6C AF700 (HK1.4, 1:300, BioLegend) and SiglecF PE-CF594 (E50-2440, 1:1000, BD Biosciences). Cells were then washed in PBS, fixed with 4% paraformaldehyde (Santa Cruz Biotechnology, Dallas, TX, USA), and strained through a 70 μm filter (BD biosciences). Data was acquired on a BD Symphony flow cytometer (BD Biosciences) using BD FACSDiva software (BD Biosciences) and analyzed using FlowJo software (v10.7.1, BD).

### scRNA-seq libraries preparation and sequencing

For scRNAseq of BMDM, Mtb-exposed cells were infected at an MOI 2.5:1 with Mtb-GFP as described above for 4 h before washing away extracellular bacteria with PBS and incubating with fresh BMM media for 8 h. PIM6-stimulated BMMs were stimulated with 1 μg/mL PIM6 for 8 h. After 8 h, cells were detached with 1% BSA in PBS at 4°C and incubated with Total-Seq B murine hashtagging (HTO) antibodies (BioLegend, number 155831, number 155833, number 155835, number 155837, number 155839, number 155841) for 30 min on ice. Cells were washed three times with 1% BSA in PBS, counted in Trypan Blue using a Countess (Thermo Fisher), and pooled. The pooled sample was centrifuged and resuspended in PBS, filtered using a Flowmi 40-µm cell strainer (Bel-Art, H13680-0040), and counted for final concentration determination and viability for 10× loading. Cells were loaded following the 10× Chromium NextGEM Single Cell 3′ v3.1 protocol with Feature Barcoding (Revision D) with the addition of 0.5 U/μL RNase inhibitor (Roche, Rotkreuz, Switzerland) to the single-cell suspension. Post GEM-RT was performed following the 10× protocol (CG000206 Rev D) through cDNA amplification at which point the cDNA was inactivated at 95°C for 15 min and removed from the BSL3 facility. Library construction for both the gene expression and HTO was then performed according to the 10× protocol. Libraries were sequenced on a NextSeq500 (Illumina, San Diego, CA, USA).

For scRNAseq of murine lung cells, a single-cell suspension of lung cells was generated as described above. Cells were then counted prior to proceeding with MULTI-seq barcoding. Samples were multiplexed as previously described. In brief, samples were barcoded with 2.5 μM of the LMO anchor and barcode for 5 min on ice in PBS before adding 2.5 μM of the LMO co-anchor and incubating for an additional 5 min. Samples were quenched with 1% BSA in PBS and washed once. Samples were processed using the 10× Genomics NextGEM Single Cell 3′ kit v3.1 per the manufacturer’s protocol in two microfluidic lanes. Again, 0.5 U/μL RNase inhibitor (Roche, Rotkreuz, Switzerland) was added to the single-cell suspension, and cDNA was inactivated at 95°C for 15 min prior to BSL3 removal. Libraries were sequenced on a NextSeq500 (Illumina, San Diego, CA USA). The data were aligned to the mm10 reference using Cell Ranger Count v6.0.1.

### scRNA-seq data processing and analysis

For BMDM scRNAseq, raw sequencing reads were converted to FASTQ files, aligned to the murine genome, and filtered, and barcodes and UMIs were counted using CellRanger (v4.0.0) from 10× Genomics. Downstream analysis then proceeded using Seurat (v3.9.9) (62) and scTransform (v 0.3.2) (63) for linear dimensional reduction. Sample identities were assigned using the HTO reads, and filtering was performed to remove doublets, cells with >25% mitochondrial reads, and cells with <250 unique genes/cell. Cells were clustered by a Shared Nearest Neighbor graph. Gene sets for principal components 1 and 2 of the single-cell dataset are composed of genes with the top 50 positive loadings for each component. The cluster 7 gene set is composed of the top 50 genes ranked by average log2 fold change identified as markers of this cluster over all others. Cells scoring >1 for each gene set are considered expressing the gene set. For overlap of gene set expression, cells scoring >1 for both gene sets are considered co-expressing cells. Functional analysis was performed using IPA (QIAGEN Inc., Germantown, MD, USA; https://www.qiagenbio-informatics.com/products/ingenuity-pathway-analysis).

For murine lung scRNAseq, LMO barcode and gene expression count matrices were merged and analyzed using R (v4.0.3) and Seurat (v4.0.0). Samples were demultiplexed using HTODemux (Seurat). Genes with high ambient RNA contribution were identified using estimateAmbience (DropletUtils) and removed from downstream analysis. Cells with less than 300 genes were detected, and more than 10% mitochondrial UMIs were excluded. Three thousand variable features were used for PCA. Counts were normalized using the default parameters from NormalizeData (Seurat), i.e., scaling by 10,000 and log normalization. Walktrap (igraph) clustering was performed on the shared nearest neighbor graph generated from FindNeighbors (Seurat) using 30 principal components and *k* = 20. Cell type annotation was based on expert annotation and predicted cell type labels from the Tabula Muris dataset. Cell-type labels were predicted using FindTransferAnchors, MappingScore, and TransferData (Seurat) with 30 dimensions and 20 trees. Lymphocyte and myeloid cell types were subclustered separately by repeating the steps above on the cell subsets. Enrichment analyses were performed using EnrichR with the GO Biological Process 2021, ChEA 2016, and MSigDB Hallmark databases. Marker gene statistics were calculated using wilcoxauc (presto). All signature scores were calculated using AddModuleScore_UCell (UCell). Differential cell type abundance analysis was performed using a generalized binomial linear model (stats, emmeans). Cell counts per cell type were modeled as a function of an interaction term describing cell type and condition.

### Statistics

Statistical tests used for each experiment are indicated in the figure legends. Two-tailed unpaired *t-*tests were used to analyze qPCR and flow cytometry data. Statistical methods for the analysis of RNAseq and scRNAseq data are included in the methods specific to those approaches; significance scores were corrected for multiple comparisons.

## Data Availability

The data sets supporting the conclusions of this study are available in the Gene Expression Omnibus: bulk RNAseq data GEO accession number GSE227851; in vitro scRNAseq GEO accession number GSE232320; mouse scRNAseq GEO accession number GSE229190. Analysis code for murine lung scRNAseq is available publicly. Analysis code for BMDM scRNAseq is available publicly.

## SUPPLEMENTAL MATERIAL

Figure S1 msystems.00052-23-s0001.eps Expression of *Tnf*.

Figure S2 msystems.00052-23-s0002.eps Contribution of each principle component to variance.

Figure S3 msystems.00052-23-s0003.eps Schematic of the established paradigm for key molecular drivers of the macrophage NF-kB and type I IFN responses to Mtb infection.

Figure S4 msystems.00052-23-s0004.eps Principal component analysis of single cell RNAseq.

Figure S5 msystems.00052-23-s0005.eps PrimeFlow experiments.

Figure S6 msystems.00052-23-s0006.eps Flow cytometric analysis of mouse lungs.

Figure S7 msystems.00052-23-s0007.tif Number of cells analyzed per condition, UMAP embedding of cell type annotations, top marker gene shown for each subset based on AUROC values, UMAP projection of cells identified in Pisu et al. on our identified cellular subsets, comparison of cellular classification between Pisu et al. and our data set, Tlr2 expression in WT mice, and fractional abundance of each cell type.

Legends msystems.00052-23-s0008.docx Supplemental figure and table legends.

Table S1 msystems.00052-23-s0009.xlsx Bulk RNAseq- expression of genes in each cluster.

Table S2 msystems.00052-23-s0010.xlsx scRNAseq gene sets.

ASM does not own the copyrights to Supplemental Material that may be linked to, or accessed through, an article. The authors have granted ASM a non-exclusive, world-wide license to publish the Supplemental Material files. Please contact the corresponding author directly for reuse.
