# Supplementary figures and images for: TLR2 is non-redundant in the population and subpopulation responses to Mycobacterium tuberculosis in macrophages and in vivo
Source: mSystems. 2023 Jul 13;8(4):e00052-23. doi: 10.1128/msystems.00052-23 (PMC10506474; doi:10.1128/msystems.00052-23)

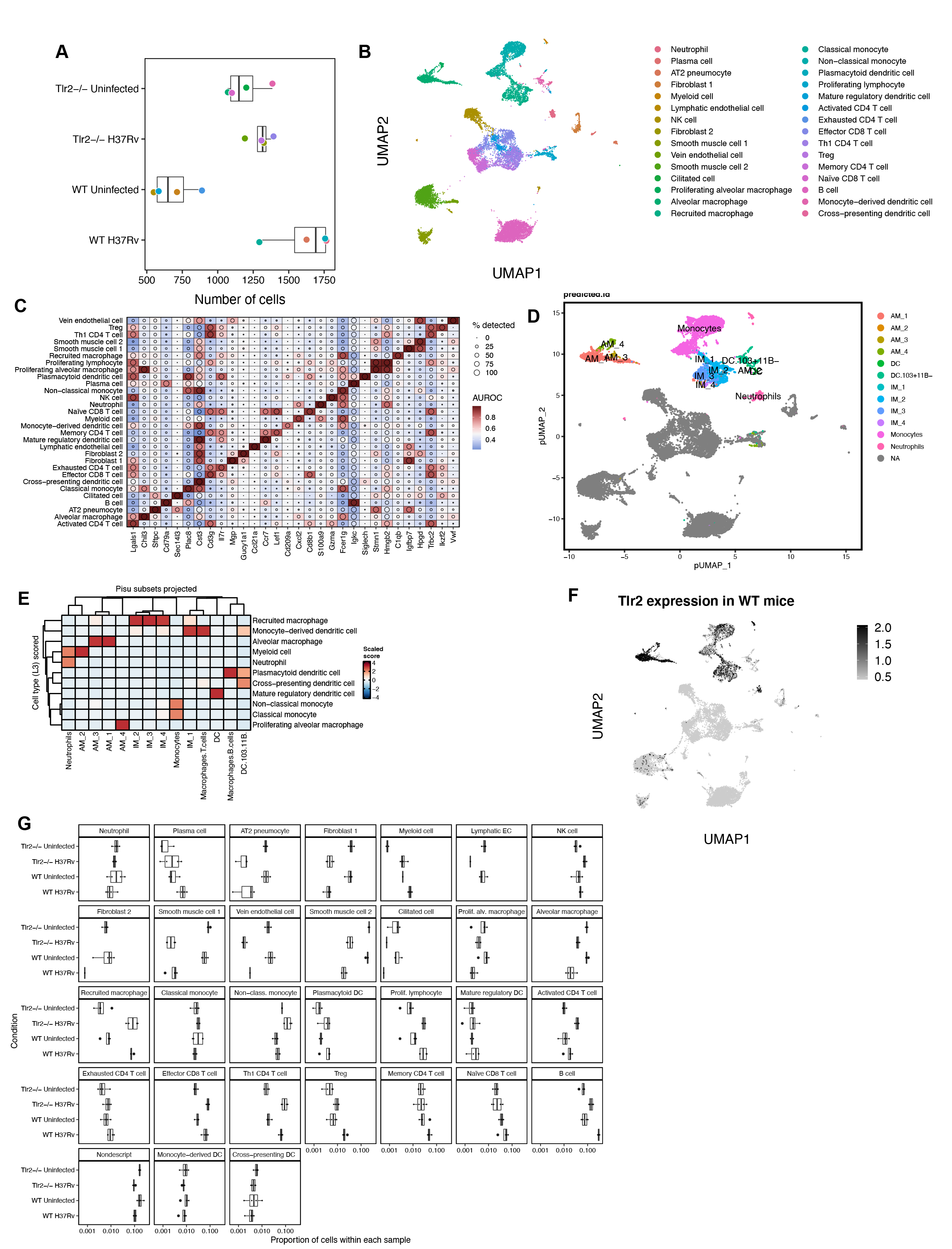

Supplement: Figure S7 — Number of cells analyzed per condition, UMAP embedding of cell type annotations, top marker gene shown for each subset based on AUROC values, UMAP projection of cells identified in Pisu et al. on our identified cellular subsets, comparison of cellular classification between Pisu et al. and our data set, Tlr2 expression in WT mice, and fractional abundance of each cell type. [file msystems.00052-23-s0007.tif]
