# Supplementary material for: TLR2 is non-redundant in the population and subpopulation responses to Mycobacterium tuberculosis in macrophages and in vivo
Source: mSystems. 2023 Jul 13;8(4):e00052-23. doi: 10.1128/msystems.00052-23 (PMC10506474; doi:10.1128/msystems.00052-23)
Supplement: Legends — Supplemental figure and table legends. [file msystems.00052-23-s0008.docx]

**Supplementary Figure Legends**

**Supplementary Figure S1. (**A) THP1 cells were PMA-stimulated, then treated with increasing concentrations of each PAMP as indicated. RNA was harvested 8 hours post-treatment, and expression of TNF was profiled by qPCR relative to GAPDH control. (B) THP1 cells were PMA-stimulated and treated with 10ug/ml of each PAMP. RNA was harvested at the indicated timepoints and expression of TNF was profiled by qPCR relative to GAPDH control. (C) THP1 cells were PMA-stimulated, then treated with each PAMP at 10ug/ml. RNA was harvested at 8 hours post-treatment for RNAseq. Heatmap showing hierarchical clustering of genes significantly altered (LFC> 2, q-value < 0.05) by each stimulus compared to the untreated samples. (D) THP1 cells were PMA differentiated and infected with GFP-expressing Mtb. Cells were then sorted into GFP- (bystander) and GFP+ (infected) populations for RNAseq. Gating strategy and relative proportions for infected and bystander populations is shown. (E) Correlation matrix for heatmap shown in Fig. 1B.

**Supplementary Figure S2.** (A) Contribution of each principle component (1-8) to variance (B) overlap of b-PC1 and b-PC2 genes with genes in Mtb/PAMP and Mtb-only cluster genes. (C) The indicated BMDM were infected with H37Rv at an MOI of 5:1; RNA was harvested 24 hours post-infection. (D-E) C57BL6 BMDM were treated with LPS at the indicated concentrations (D) or infected with SL1344 at the indicated MOI (E) and RNA was harvested at the indicated timepoints. (C-E) Gene expression was quantified relative to GAPDH control. Mean +/- SD.

**Supplementary Figure S3.** (A) Schematic of the established paradigm for key molecular drivers of the macrophage NF-kB and type I IFN responses to Mtb infection. (B-C) C57BL6 BMDM (B) or the indicated BMDM (C) were treated with PIM6 1ug/ml, and RNA was harvested at the indicated timepoints post-treatment (B) or 2 hours post-treatment (C). Expression of the indicated genes was profiled by qPCR relative to GAPDH control. Mean +/- SD for 4 replicates. Graphic in A made using BioRender.

**Supplementary Figure S4.** (A) Principal component analysis of single cell RNAseq. (B) Contribution of each principal component (1-30) (C) Overlap between top 50 genes contributing to PC1, PC2, and other dominant PCs (D-E) Expression of top genes contributing to s-PC1 (D) and s-PC2 (E) in individual cells across all conditions.

**Supplementary Figure S5.** (A) THP1 cells were infected with Mtb-GFP and either paraformaldehyde fixed (top) or processed according to our optimized PrimeFlow protocol (bottom). (B) Gating strategy for identifying bystander (GFP-) and infected (GFP+) cells in PrimeFlow experiments. (C) PrimeFlow staining of homogeneously expressed control gene Rpl13.

**Supplementary Figure S6.** (A) C57BL/6J and TLR2-/- mice were infected with H37Rv via low-dose aerosol infection. Mouse lungs were harvested day 0 and plated for CFU to establish inoculum and day 42 for CFU and generation of single-cell suspensions for flow cytometry or single-cell RNAseq. Mean +/- SD for 3-5 replicates. (B) Flow cytometric analysis of mouse lungs was performed to identify major immune cell populations. An analytic scheme was developed based on a published protocol (Yu *et al*, 2016). Contour plots and gating strategy to identify macrophage subpopulations, T cells, B cells, eosinophils, and neutrophils is shown for a representative example of C57BL/6J mouse infected with H37Rv.

**Supplementary Figure S7.** (A) Number of cells analyzed per condition across 4 individual replicates (2 biological replicates and 2 technical replicates). (B) UMAP embedding of cell type annotations. (C) Top marker gene shown for each subset based on AUROC values. AUROC values and percentage of cells with each gene detected shown. (D) UMAP projection of cells identified in Pisu et al on our identified cellular subsets (E) Comparison of cellular classification between Pisu et al and our dataset (F) Average Tlr2 expression in WT mice projected on UMAP embedding. (G) Fractional abundance of each cell type within each sample shown across conditions. Abbreviations: EC- endothelial cell; NK- natural killer; DC- dendritic cell.

**Supplementary Table Legends**

**Supplementary Table S1. Bulk RNAseq and principal component analysis (PCA) of bulk RNAseq data- expression of genes in each cluster, PCA loadings, PCA scores, and PCA explained variance.**

**Supplementary Table S2. scRNAseq of BMDM- expression of genes contributing to each cluster.**
